# Post–Intensive Care Syndrome and Caregiver Burden: A Post Hoc Analysis of a Randomized Clinical Trial

**DOI:** 10.1001/jamanetworkopen.2025.3443

**Published:** 2025-04-08

**Authors:** Soojung Ahn, Marianna LaNoue, Han Su, Amanda C. Moale, Leslie P. Scheunemann, Amy L. Kiehl, Ivor S. Douglas, Matthew C. Exline, Michelle N. Gong, Babar A. Khan, Robert L. Owens, Margaret A. Pisani, Peter Rock, James C. Jackson, E. Wesley Ely, Timothy D. Girard, Leanne M. Boehm

**Affiliations:** 1 Connell School of Nursing, Boston College, Chestnut Hill, Massachusetts; 2 School of Nursing, Vanderbilt University, Nashville, Tennessee; 3 Critical Illness, Brain dysfunction, and Survivorship Center at Vanderbilt, Nashville, Tennessee; 4 Department of Medicine, Division of Pulmonary, Allergy, Critical Care, and Sleep Medicine, University of Pittsburgh, Pittsburgh, Pennsylvania; 5 Department of Medicine, Divisions of Geriatrics and Pulmonary, Allergy, Critical Care, and Sleep Medicine, University of Pittsburgh, Pittsburgh, Pennsylvania; 6 Department of Medicine, Division of Allergy, Pulmonary and Critical Care Medicine, Vanderbilt University Medical Center, Nashville, Tennessee; 7 Denver Health Department of Medicine, Pulmonary Sciences and Critical Care Medicine, University of Colorado, Anschutz MC, Denver; 8 Department of Internal Medicine, Division of Pulmonary, Critical Care, and Sleep Medicine, Ohio State University, Columbus; 9 Division of Critical Care Medicine, Division of Pulmonary Medicine, Department of Medicine, Montefiore Healthcare System/Albert Einstein College of Medicine, Bronx, New York; 10 Division of Pulmonary, Critical Care, Sleep and Occupational Medicine, Department of Medicine, Indiana University School of Medicine, Indianapolis; 11 Division of Pulmonary, Critical Care and Sleep Medicine, University of California San Diego, La Jolla; 12 Division of Pulmonary, Critical Care and Sleep Medicine, Department of Medicine, Yale University School of Medicine, New Haven, Connecticut; 13 Department of Anesthesiology, University of Maryland School of Medicine, Baltimore; 14 Geriatric Research Education and Clinical Center, Tennessee Valley Veterans Affairs, Nashville; 15 Veterans Affairs Tennessee Valley Health System Geriatric Research, Education, and Clinical Center (GRECC), Nashville; 16 Center for Research, Investigation, and Systems Modeling of Acute Illness in the Department of Critical Care Medicine, University of Pittsburgh School of Medicine, Pittsburgh, Pennsylvania

## Abstract

**Importance:**

Understanding the reciprocal association between post–intensive care syndrome (PICS) and caregiver burden is crucial for optimal care of patients and caregivers following critical illness.

**Objective:**

To evaluate the associations between patient post–intensive care impairments and caregiver burden.

**Design, Setting, and Participants:**

This secondary analysis of the MIND-USA study, a multicenter randomized clinical trial, which enrolled patients admitted to intensive care units (ICU) from 16 academic medical centers across the US (December 2011 to August 2017), included 148 patient-caregiver dyads. Patients were adults aged 18 years or older with ICU delirium randomized to receive haloperidol, ziprasidone, or placebo. A caregiver who provided unpaid assistance to the patient was identified at enrollment. PICS and caregiver burden were assessed at 3 months and 12 months after randomization. Statistical analysis was performed from March 2023 to April 2024.

**Main Outcomes and Measures:**

ICU survivors were assessed for PICS domains, including physical and cognitive function, and posttraumatic stress disorder using the Katz Activities of Daily Living, the Functional Activities Questionnaire, the Telephone Interview for Cognitive Status, and the Posttraumatic Stress Disorder Checklist-Civilian version, respectively. Caregiver burden was assessed using the Zarit Burden Interview. The associations between patient PICS and caregiver burden at 3 and 12 months were examined using structural equation modeling.

**Results:**

Of 148 patients included in this study with a median (IQR) age of 58 (48-65) years, the majority identified as male (79 patients [53.4%]), and there were 16 (10.8%) Black, 139 (93.9%) non-Hispanic, and 127 (85.8%) White patients. PICS and caregiver burden at 3-month follow-up was positively associated with these outcomes at 12-month follow-up (PICS: β = 0.69; 95% CI, 0.50 to 0.88; *P* < .001; caregiver burden: β = 0.68; 95% CI, 0.53 to 0.82; *P* < .001). However, contrary to the study hypotheses, significant associations between 3-month PICS and 12-month caregiver burden and between 3-month caregiver burden and 12-month PICS were not observed (PICS→caregiver burden: β = 0.82; 95% CI, −0.02 to 1.66; *P* = .09; caregiver burden→PICS: β = 0.00; 95% CI, −0.03 to 0.03; *P* = .95). There was significant covariance between PICS and caregiver burden at each time point.

**Conclusions and Relevance:**

In this secondary analysis of a randomized clinical trial of ICU survivors and their caregivers, patient PICS and caregiver burden were associated at concurrent time points but were not associated with each other longitudinally.

**Trial Registration:**

ClinicalTrials.gov Identifier: NCT01211522

## Key Points

**Question:**

What is the long-term association between patient post–intensive care syndrome (PICS) and caregiver burden following discharge from the intensive care unit (ICU)?

**Findings:**

Among 148 ICU survivors who had incident delirium and their caregivers in this secondary analysis of a randomized clinical trial, significant correlations were found between patient PICS and caregiver burden at both 3-month and 12-month follow-ups. However, no longitudinal reciprocal associations were observed between patient PICS and caregiver burden at these time points.

**Meaning:**

These data suggest that while PICS and caregiver burden are correlated at specific time points, they may not have a long-term reciprocal association with each other.

## Teaser

This secondary analysis of a randomized clinical trial evaluates the associations between patient post–intensive care impairments and caregiver burden.

## Introduction

After critical illness, patients often experience significant physical, cognitive, and psychological health consequences, collectively known as post–intensive care syndrome (PICS).^1^ These symptoms can profoundly impact both patients and their caregivers. Family members often find themselves unprepared for caregiving demands, with insufficient time to adapt to their new roles. A lack of preparedness can render it challenging to manage care and navigate formal support resources.^2,3,4^ Caregivers of patients with functional impairments are at heightened risk of caregiver burden, emotional distress, poor sleep, posttraumatic stress disorder (PTSD), lifestyle adjustments, and diminished health-related quality of life.^5,6,7,8,9,10,11^

In the broader context of caregiving, caregiver burden can persist throughout the patient’s recovery, adversely affecting care quality and impeding patient recovery and rehabilitation.^12,13,14^ Conversely, positive emotional functioning support from caregivers contributes to favorable rehabilitation outcomes, such as improved physical function and occupational and social integration for patients.^15,16^ This interdependence between patient health outcomes and caregiver burden may extend to critical illness and its long-term recovery. Previous studies have identified associations between patient PICS and caregiver burden. For instance, cohort studies conducted in Sweden and Spain found significant associations between patients’ physical impairment and mental health problems, such as anxiety and PTSD, and higher caregiver burden and mental health at 3 months after intensive care unit (ICU) discharge.^6,17^

However, little is known about longitudinal and reciprocal associations between PICS and caregiver burden. Given that caregiver involvement is integral to ICU survivors’ recovery and rehabilitation,^18^ further investigations are warranted to explore the potential interdependent associations. Understanding how patient impairments and caregiver burden affect each other throughout recovery can enable the development of more effective support strategies for the dyads following critical illness.

To address this gap, we examined the associations between patient PICS and caregiver burden at 3- and 12-month follow-up time points for participants in an ICU-based randomized clinical trial (RCT). We hypothesized that (1) higher levels of PICS at each time point would be associated with higher levels of caregiver burden at the same time point, (2) higher levels of PICS at 3 months would be associated with higher levels of caregiver burden at 12 months, and (3) higher levels of caregiver burden at 3 months would be associated with higher levels of PICS at 12 months.

## Methods

### Study Design, Setting, and Participants

We conducted a post hoc exploratory analysis of data from the MIND-USA study, a randomized, double-blind, and placebo-controlled trial comparing the effects of haloperidol, ziprasidone, and placebo on delirium during critical illness and subsequent long-term outcomes.^19,20^ The parent study included 566 adults (aged ≥18 years) from 16 academic medical centers across the US admitted to a medical or surgical ICU with respiratory failure and/or septic or cardiogenic shock and delirium from December 2011 to August 2017. Caregivers, identified at enrollment or follow-up, were defined as individuals providing unpaid assistance, support, or care to the patient due to illness, disability, or aging (eg, spouse, child, sibling, or significant other). Ethical approval was obtained at each participating study site, and written informed consent was obtained from each participant or an authorized representative. The parent study design, inclusion and exclusion criteria, randomization and blinding, procedures, and main results have been previously reported (trial protocol and statistical analysis plan in Supplement 1).^19,20^ The Consolidated Standards of Reporting Trials (CONSORT) reporting guidelines were followed for the parent study and this post hoc analysis.

### Measures

#### Post–Intensive Care Syndrome

In the parent study, patient participants were assessed for long-term physical (ie, disability in activities of daily living [ADL] and instrumental ADL), cognitive (ie, global cognition), and psychological outcomes (ie, PTSD), as part of secondary end points of the delirium treatment. At 3 and 12 months after randomization, trained research coordinators administered validated tests and questionnaires via telephone. The 6-item Katz Index of Independence in ADL measured patient independence in ADLs (eg, bathing and dressing) (items scored 0 [normal] to 2 [dependent]).^21^ The 10-item Functional Activities Questionnaire (FAQ) measured independence in instrumental ADLs (eg, meal preparation and finance management) (items scored 0 [normal] to 3 [dependent]).^22^ The 11-item Telephone Interview for Cognitive Status (TICS) measured orientation, attention, language, and memory.^23^ In the study, age-adjusted T scores derived from the TICS total score were used (possible range: 13-80).^24^ The 17-item PTSD Checklist-Civilian version (PCL-C) measured symptoms of PTSD (ie, intrusive reexperiencing, avoidance, and hyperarousal symptoms) in the past month, with the ICU hospitalization and any medical or surgical events occurring at that time as the referent traumatic event (items scored 1 [not at all] to 5 [extremely]).^25^

#### Caregiver Burden

Caregiver burden was measured using the Zarit Burden Interview (ZBI) with the same caregiver at 3 and 12 months. The ZBI is a 12-item measure evaluating how often caregivers experience feelings including anger, stress, loss of control, uncertainty, or other challenges because of their involvement with or caring for the patient (items scored 0 [never] to 4 [nearly always]).^26^

#### Demographic and Clinical Characteristics

We assessed patient demographic and clinical characteristics associated with PICS domains as potential covariates, including age, sex, and precritical illness health status assessed at baseline (Charlson Comorbidity Index,^27^ Informant Questionnaire on Cognitive Decline in the Elderly [IQCODE] score,^28^ and Clinical Frailty Scale^29^) based on the literature.^30^ The 16-item IQCODE assesses cognitive decline associated with functional impairment based on informant reports (scored 1 [much worse] to 5 [much better]).^28^ The Clinical Frailty Scale indicates the overall level of frailty (scored 1 [very fit] to 7 [severely frail]).^29^

### Statistical Analysis

PICS domains and caregiver burden were treated as continuous. We inspected the distributions and performed statistical assessments for departures from normality. As 3 of the 4 PICS measures (ADL, FAQ, and PCL-C) were significantly nonnormal at both time points, we used nonparametric tests for bivariate associations. We used Wilcoxon signed rank test and McNemar test to analyze changes over time in the levels of PICS and caregiver burden and the proportions of patients and caregivers meeting the thresholds. We calculated descriptive statistics for all demographic and clinical characteristics and primary study measures at each time point and examined bivariate association matrices within and across time. As these data come from a parent RCT with 3 groups across multiple sites, we first assessed for group and cluster effects for the PICS measures. As no group effect was significant, and no intraclass correlation coefficient exceeded 0.05, these effects were omitted in modeling.

While there is ongoing work to understand the nature of PICS and appropriate methods for its assessment,^31^ there is currently no criterion standard measurement framework. The literature predominantly conceptualizes PICS by combining measures of physical function, cognitive function, and mental health, using various methods such as the sum of binary indicators, stepwise processes, or algorithmic approaches.^32^ Given these methods are not empirically supported or data-driven, we elected to operationalize PICS with our available indicators using structural equation modeling (SEM). SEM represents, estimates, and tests a network of relationships between both measured variables and unobserved (latent) constructs.^33^ We first estimated measurement models for the PICS latent variable using confirmatory factor analysis at both 3 and 12 months to test the associations between all the measured PICS indicators and PICS as an underlying latent variable. We transformed the nonnormal indicators (ADL, FAQ, and PCL-C) for inclusion.

To test our hypotheses, we fitted an SEM model including the PICS measurement models and hypothesized multivariate associations. The final model included direct effects from both 3-month variables to both 12-month variables, cross-sectional covariances at 3 and 12 months, and cross-lagged indicator covariances between 3-month and 12-month PICS indicators (ADL, PCL-C, and TICS). To estimate the associations of individual-level variables with changes in patient and caregiver outcomes, we included only cases with complete data at both 3 and 12 months in the analytic sample. For the sensitivity analysis, we fitted the identical SEM model, adding cases with complete data at 3 months but missing data at 12 months.

SEM models were estimated with robust maximum likelihood estimation in the R package lavaan.^34^ Model fit was assessed using χ^2^ (where small and nonsignificant values are indicative of better fit), root mean square error of approximation (RMSEA), standardized root mean square residual (SRMR), Tucker-Lewis Index (TLI), and the comparative fit index (CFI). Good model fit is indicated by RMSEA less than 0.06, SRMR less than 0.08, and TLI and CFI greater than 0.90.^35^ Statistical significance was indicated by a 2-sided *P* value less than .05. Statistical analysis was performed in R version 4.2.3 (R Project for Statistical Computing) and SPSS version 29 (IBM) from March 2023 to April 2024.

## Results

### Participant Characteristics

**Figure 1.**
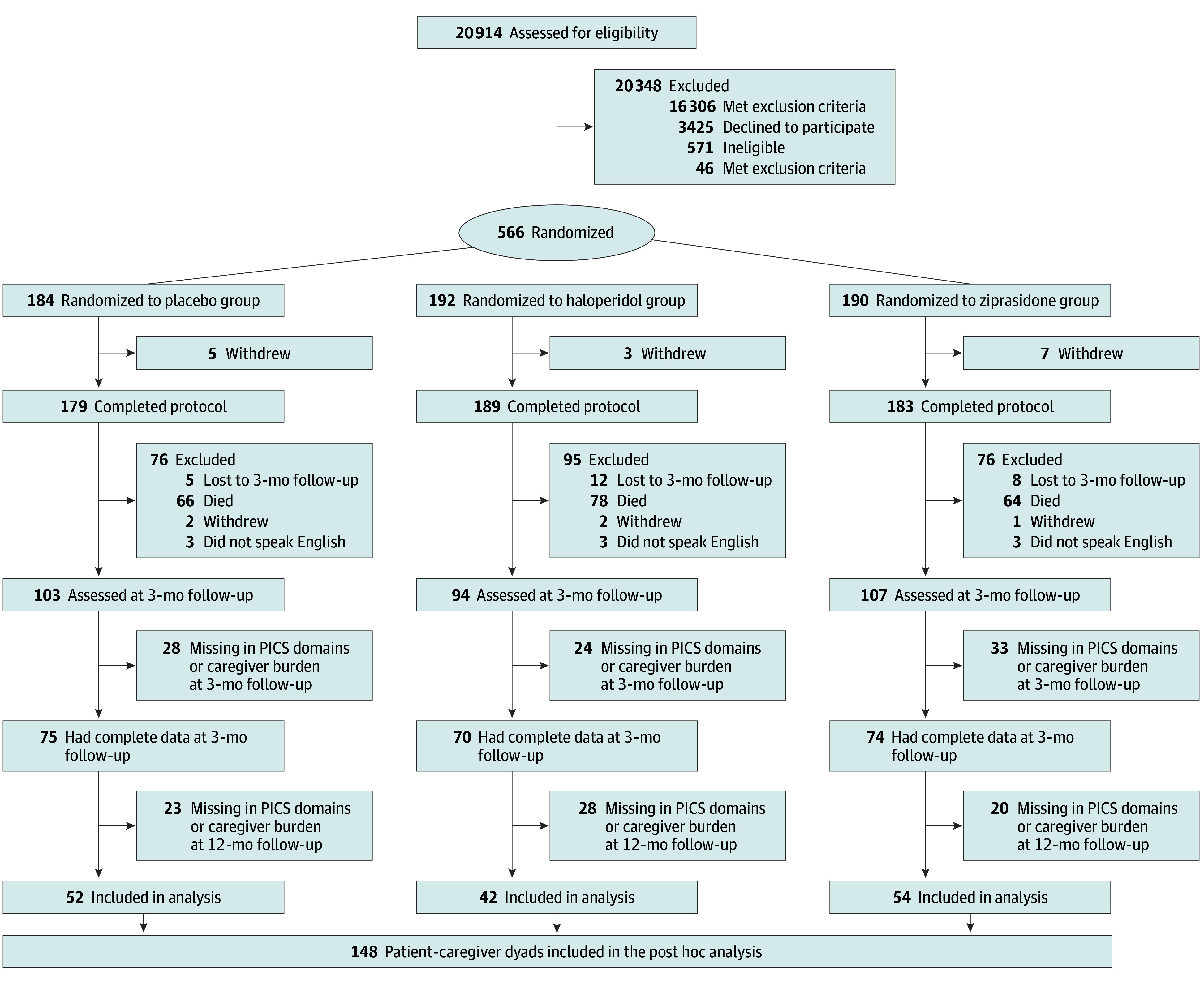
Study Flowchart for the Post Hoc Analysis

Of 148 patients included in this study with a median (IQR) age of 58 (48-65) years, the majority were male (79 patients [53.4%]), and there were 16 (10.8%) Black, 139 (93.9%) non-Hispanic, and 127 (85.8%) White patients. Most patients had a high school education or some college experience (91 patients [62.3%]). Demographic and clinical characteristics of the sample are summarized in Table 1. Figure 1 shows the sample selection flow for this study. Among the 219 patient-caregiver dyads fully followed up at 3 months in the parent study, 71 dyads (32.4%) were excluded from this analysis due to either missing follow-up data or incomplete outcome data for either the patient or caregiver at 12 months. Therefore, our analytic sample for this post hoc analysis consisted of 148 patient-caregiver dyads who had complete data for both 3- and 12-month assessments.

**Table 1.** Patient Characteristics and Caregiver Burden

Characteristic	Individuals, No. (%) (N = 148 dyads)
Patients	
Median age (IQR), y	58 (48-65)
Sex	
Male	79 (53.4)
Female	69 (46.6)
Race	
Asian	5 (3.4)
Black/African American	16 (10.8)
White	127 (85.8)
Ethnicity	
Hispanic	9 (6.1)
Non-Hispanic	139 (93.9)
Education^a^	
Less than high school	14 (9.6)
High school diploma or GED	54 (37.0)
Some college, no degree	37 (25.3)
Associate degree	16 (11.0)
Bachelor’s degree	20 (13.7)
Master’s degree	5 (3.4)
Pre-ICU Charlson Comorbidity Index score, median (IQR)^b^	2 (0-3)
Pre-ICU IQCODE score, median (IQR)^c^	3 (3-3.2)
Pre-ICU Frailty score, median (IQR)^d^	2 (2-3)
ADL, median (IQR)^e^	
3 mo	0 (0-2)
12 mo	0 (0-1.8)
FAQ, median (IQR)^f^	
3 mo	4 (0-9)
12 mo	3 (0-7)
TICS [age-adjusted], median (IQR)^g^	
3 mo	42 (33-51)
12 mo	46 (33-55)
PCL-C, median (IQR)^h^	
3 mo	26 (21-36.8)
12 mo	27 (21.3-35)
Caregivers	
Caregiver burden, median (IQR)^i^	
3 mo	11 (6-17)
12 mo	8 (3-14.8)

Abbreviation: ADL, Katz Index of Activities of Daily Living; FAQ, Functional Activities Questionnaire; ICU, intensive care unit; IQCODE, Informant Questionnaire on Cognitive Decline in the Elderly; PCL-C, Posttraumatic Stress Disorder Checklist-Civilian version; TICS, Telephone Screening of Cognitive Status.

^a^ Two missing.

^b^ Charlson comorbidity index scores range from 0 to 33, with higher scores indicating a greater burden of chronic illness.

^c^ IQCODE scores range from 1 to 5, with a score of 3 indicating no change in cognition over the past 10 years. Scores lower than 3 indicate improvement while scores greater than 3 indicate decline.

^d^ Frailty scores range from 1 to 7, with higher scores indicating more severe frailty.

^e^ ADL scores range from 0 to 12 (higher scores indicate greater dependence; ≥1 indicates disability).

^f^ FAQ scores range from 0 to 30 (higher scores indicate greater dependence; ≥9 indicates impaired function).

^g^ Age-adjusted TICS scores range from 13 to 80 points (higher scores indicate better cognitive function; ≤35 indicates cognitive impairment).

^h^ PCL-C scores range from 17 to 85 (higher scores indicate more severe posttraumatic stress disorder symptoms; ≥38 indicate diagnosis of PTSD).

^i^ Caregiver burden scores range from 0 to 48 (higher scores indicate greater burden).

### Descriptive Analysis of PICS and Caregiver Burden

There were no significant changes over time in the continuous measures of PICS domains. Across the PICS domains, impairments were noted as follows: 65 patients (43.9%) in ADL (≥1),^21^ 39 patients (26.4%) in FAQ (≥9),^22^ 41 patients (27.7%) in TICS (≤35),^20^ and 36 patients (24.3%) in PCL-C (≥38)^25^ at 3 months. The proportions were 57 patients (38.5%), 30 patients (20.3%), 41 patients (27.7%), and 32 patients (21.6%), respectively, at 12 months. There was no statistically significant difference in the proportion of PICS impairments between 3- and 12-month follow-ups. The mean caregiver burden score at 12 months decreased compared with the 3-month time point (mean [SD] difference, 1.87 [6.65]; *Z* = −2.85; *P* < .001). Based on the suggested cutoff point (≥17),^26^ 41 caregivers (27.7%) reported high burden at 3 months and 31 (20.9%) reported high burden at 12 months; yet the difference in the proportions was not statistically significant.

### Bivariate Associations

Table 2 shows the bivariate associations among all measured variables within and across time. Among the PICS indicators at 3 months, most intercorrelations were significant, and all indicators except for the TICS were correlated with caregiver burden at the same time point. Among the 12-month PICS indicators, all variables showed significant intercorrelations except for between TICS and PCL-C, and all were correlated with 12-month caregiver burden except for TICS.

**Table 2.** Bivariate Associations of Post–Intensive Care Syndrome (PICS) Domains and Caregiver Burden at 3 and 12 Months^a^

Outcome	Association, ρ											
	ADL, 3 mo	FAQ, 3 mo	TICS, 3 mo	PCL, 3 mo	CB, 3 mo	ADL, 12 mo	FAQ, 12 mo	TICS, 12 mo	PCL, 12 mo	CB, 12 mo	Age	Sex, *t*
ADL, 3 mo	1.00	NR	NR	NR	NR	NR	NR	NR	NR	NR	NR	NR
FAQ, 3 mo	0.59	1.00	NR	NR	NR	NR	NR	NR	NR	NR	NR	NR
* P* value	<.001	NA	NA	NA	NA	NA	NA	NA	NA	NA	NA	NA
TICS, 3 mo	−0.17	−0.18	1.00	NR	NR	NR	NR	NR	NR	NR	NR	NR
* P* value	.04	.03	NA	NA	NA	NA	NA	NA	NA	NA	NA	NA
PCL, 3 mo	0.21	0.30	0.01	1.00	NR	NR	NR	NR	NR	NR	NR	NR
* P* value	.01	<.001	.90	NA	NA	NA	NA	NA	NA	NA	NA	NA
CB, 3 mo	0.18	0.33	0.05	0.19	1.00	NR	NR	NR	NR	NR	NR	NR
* P* value	.03	<.001	.52	.02	NA	NA	NA	NA	NA	NA	NA	NA
ADL, 12 mo	0.62	0.46	−0.14	0.23	0.15	1.00	NR	NR	NR	NR	NR	NR
* P* value	<.001	<.001	.09	.01	.07	NA	NA	NA	NA	NA	NA	NA
FAQ, 12 mo	0.49	0.53	−0.30	0.27	0.19	0.65	1.00	NR	NR	NR	NR	NR
* P* value	<.001	<.001	<.001	.001	.02	<.001	NA	NA	NA	NA	NA	NA
TICS, 12 mo	−0.19	−0.18	0.64	−0.05	0.09	−0.19	−0.35	1.00	NR	NR	NR	NR
* P* value	.02	.03	<.001	.57	.32	.02	<.001	NA	NA	NA	NA	NA
PCL, 12 mo	0.25	0.19	−0.13	0.65	0.09	0.36	0.37	−0.14	1.00	NR	NR	NR
* P* value	.002	.02	.11	<.001	.27	<.001	<.001	.09	NA	NA	NA	NA
CB, 12 mo	0.28	0.23	0.09	0.23	0.63	0.31	0.27	0.05	0.26	1.00	NR	NR
* P* value	<.001	.01	.28	.004	<.001	<.001	<.001	.59	.001	NA	NA	NA
Age	0.10	0.09	−0.14	−0.43	−0.17	0.11	0.05	−0.12	−0.35	−0.16	1.00	NR
* P* value	.25	.26	.09	<.001	.04	.20	.53	.16	<.001	.05	NA	NA
Sex, *t*	0.56	0.05	0.03	−0.92	−1.66	1.06	0.59	−1.00	−0.18	−1.37	1.05	1.00
* P* value	.58	.96	.98	.36	.10	.29	.56	.32	.86	.17	.30	NA
Frailty	0.25	0.28	−0.07	0.03	−0.01	0.21	0.20	−0.13	−0.02	0.10	0.25	*t*, 1.75
* P* value	.002	<.001	.43	.72	.91	.01	.01	.11	.81	.23	.002	.08

Abbreviations: ADL, Katz Index of Activities of Daily Living; CB, caregiver burden; FAQ, Functional Activities Questionnaire; NA, not applicable; NR, not reported; PCL, Posttraumatic Stress Disorder Checklist-Civilian version; TICS, Telephone Screening of Cognitive Status.

^a^ Coefficients (*P* value) are Spearman ρ for continuous-to-continuous associations and *t* (*P* value) for between-group (row for sex and cell where sex and frailty intersect).

All the 3-month PICS indicators except the TICS were significantly correlated with 12-month caregiver burden. The 3-month caregiver burden was significantly correlated with 12-month FAQ, yet not with ADL, TICS, or PCL-C. Additionally, the level of frailty was correlated with ADL and FAQ at both time points. Based on the patterns of bivariate associations among the variables known to be associated with PICS and the primary study variables (eTables 1 and 2 in Supplement 2), and considering the limitation of the small sample size, frailty was included in the model solely as a covariate.

### Measurement Model Fit

**Figure 2.**
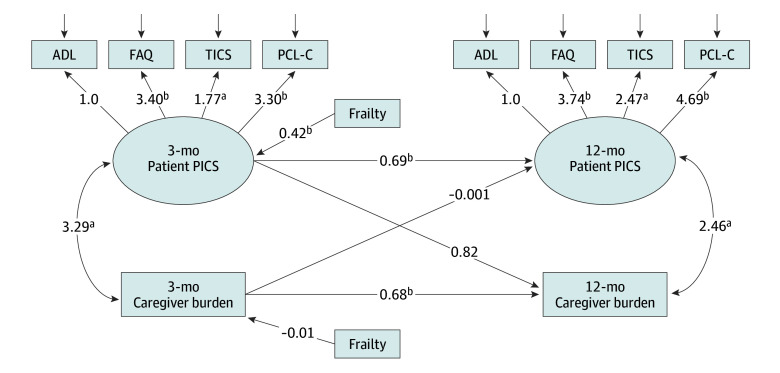
Structural Equation Modeling of Associations Between Post–Intensive Care Syndrome and Caregiver Burden Coefficients of pathways are shown in the figure. Latent variables are represented by ovals and observed variables by rectangles. The values in the top panel where arrows from latent variables point to observed variables represent factor loadings derived from confirmatory factor analysis. The curved arrow represents covariance between the variables. The model was adjusted for patient frailty. ADL indicates activities of daily living; FAQ, Functional Activities Questionnaire; PCL-C, Posttraumatic Stress Disorder Checklist-Civilian version; TICS, Telephone Interview for Cognitive Status; PICS, post–intensive care syndrome. ^a^*P* < .01. ^b^*P* < .001.

The measurement model demonstrated that the observed PICS indicator data adequately represented the underlying structure of the PICS latent variable at both time points (Figure 2) (3-month model: RMSEA, 0.00; 95% CI, 0.00-0.12; χ^2^_2_ = 0.76; SRMR, 0.02; TLI, 1.05; CFI, 1.0; 12-month model: RMSEA, 0.10; 95% CI, 0.00-0.22; χ^2^_2_ = 5.29; SRMR, 0.04; TLI, 0.92; CFI, 0.97).

### Structural Equation Modeling

The model fitting indices confirmed the estimated model was well supported by the data (χ^2^_36_ = 45.38; *P* = .14; RMSEA, 0.04; 95% CI, 0.00 to 0.08; SRMR, 0.06; CFI, 0.98; TLI, 0.97). As hypothesized, 3-month PICS (latent) and caregiver burden were significantly associated with these outcomes at 12 months (PICS: β = 0.69; 95% CI, 0.50 to 0.88; *P* < .001; caregiver burden: β = 0.68; 95% CI, 0.53 to 0.82; *P* < .001) (Figure 2). Contrary to our hypotheses, the associations between 3-month PICS and 12-month caregiver burden and between 3-month caregiver burden and 12-month PICS were not significant (PICS→caregiver burden: β = 0.82; 95% CI, −0.02 to 1.66; *P* = .09; caregiver burden→PICS: β = 0.00; 95% CI, −0.03 to 0.03; *P* = .95) (Figure 2). There was a significant correlation between PICS and caregiver burden at each time point.

### Sensitivity Analysis

When we re-estimated the measurement model in the sample with complete data at 3 months (219 patient-caregiver dyads), the model fit comparably well (RMSEA <0.01; 95% CI, 0.00-0.05; χ^2^_2_ = 0.22; *P* = .90; CFI, 1.0; SRMR, 0.01). The structural model of the cross-sectional association between the PICS latent variable and caregiver burden also had good fit (χ^2^_5_ = 3.13; *P* = .68; RMSEA, 0.00; CFI, 1.0; SRMR, 0.04). The parameter estimate for the relationship between PICS and caregiver burden at 3 months (β = 2.72; 95% CI, 1.53-3.92; *P* < .001) was comparable with that in the full model.

## Discussion

In our post hoc exploratory analysis of a multicenter RCT, we assessed the reciprocal association between patient PICS and caregiver burden using SEM. While we did not observe longitudinal relationships between latent PICS and caregiver burden, we found significant associations within each time point, which is aligned with previous findings.^6,17^

Our findings revealed significant cross-sectional but not longitudinal associations between PICS and caregiver burden, suggesting a short-term association that does not persist to 12 months. While overall PICS scores did not correlate with longitudinal caregiver burden, individual domains related to ADL/IADL and PTSD did. Specifically, ICU survivors’ physical impairments consistently contributed to caregiver burden over time, with significant correlations between ADL/IADL at 3 months and caregiver burden at 12 months. The physical demands and time investment required to support patients with ADL/IADL limitations, along with their higher medical needs, may explain this association. Caregivers often assume demanding roles after ICU discharge without adequate preparation, significantly amplifying burden.^36,37^ Despite practice guidelines for family engagement in patient care from ICU admission to improve caregiver readiness, there remains no consensus on effective engagement strategies.^38,39,40,41^ Additionally, while patients and families typically receive home health and rehabilitation services in the initial weeks to months postdischarge, coverage often becomes limited despite ongoing impairments. Effective integration of comprehensive caregiver assessments into routine clinical practice to tailor caregiver preparation for supporting patients’ physical and psychological functioning, along with access to longitudinal caregiver resources, may alleviate burden.

While up to 30% of survivors experience post-ICU impairment at 6 months,^42^ caregiver burden and emotional effects of critical illness (ie, PICS-family) can persist for years.^43^ Our study found that PICS and caregiver burden at 3 months were associated with these outcomes at 12 months, with no linear improvement over time. This finding underscores the critical need for early and longitudinal assessment of caregiver needs and health outcomes to provide timely resources, including financial, social, and mental health support.^44^ Caregiver experiences are influenced by various contextual factors such as the patient-caregiver relationship, socioeconomic status, and cultural context.^45,46,47,48^ Therefore, it is imperative to develop support approaches tailored to the unique needs of each individual. By considering the ICU survivor-caregiver as a unit of care, these strategies can help mitigate the long-term repercussions of critical illness on caregivers while supporting patient recovery. Given the infancy of research on the complexity of patient and caregiver outcomes, further studies are warranted to deepen our understanding of post-ICU caregiving and its role in ICU survivorship.

While early postdischarge caregiver burden was not associated with long-term PICS in our study, broader caregiving literature suggests that other caregiver factors may influence patient health and rehabilitation outcomes. For example, positive caregiver involvement in poststroke care enhances patient participation in rehabilitation and improves physical function,^15,49,50^ while caregiver preparedness and confidence in heart failure improves patient self-care.^51,52^ However, little is known about caregiver factors influencing recovery trajectories of ICU survivors. Prospective evaluation of caregiver health and its impact on patient outcomes, including care quality, patient recovery, and readmission rates will advance our understanding of the reciprocal effects between patient and caregiver outcomes and support policy changes and interventions to better support caregivers throughout the patient’s recovery.

### Strengths and Limitations

Our study has several strengths. We used longitudinal data for both patients and their caregivers. By including data from 3- and 12-month follow-up time points, we reduced potential confounding effects that could result from a between-group study design due to interindividual differences. Additionally, using SEM allowed us to include multiple indicators of the PICS construct in our model simultaneously while accounting for measurement error.^53^

Our study has limitations inherent to post hoc analysis of an RCT. First, our analytic sample included only a small proportion of patients and caregivers from the parent study with complete data at both time points, which might have introduced bias. The missing values may not be random, and many losses to 12-month follow-up were likely due to patient death and severe medical conditions, leading overwhelmed caregivers to refuse follow-up, potentially excluding the highest burden patients and caregivers. The small sample size also limited our ability to explore confounding factors, reduced statistical power, and affected generalizability.^54^ Second, our sample characteristic may limit the generalizability of the findings. Our analysis uniquely evaluated post-ICU trajectories in patients who all experienced ICU delirium. Given that many patients with ICU delirium have preexisting neurocognitive deficits^55,56,57^ and caregiver adjustment may precede the critical illness, the lack of cognitive function manifestation as PICS and its minimal contribution to caregiver burden may be due to our sample characteristics. Further studies are needed to replicate our findings in other ICU survivor and caregiver cohorts. Third, our PICS measures do not align with current recommendations. The parent study, designed before the publication of PICS stakeholder consensus, solely used patient-reported measures, potentially introducing response bias. Although the core outcome set for PICS is yet undefined, it is recommended to use both patient-reported and performance-based measurements (eg, timed up-and-go and handgrip strength).^31,58^ Additionally, the parent study did not include measures for depression and anxiety for long-term psychological outcomes. Therefore, our PICS latent variable lacks these indicators. Fourth, information on caregiver characteristics was not available in the parent study. Various caregiver factors (eg, age, gender, race, ethnicity, socioeconomic status, health, preparedness for caregiving, and medical conditions) and caregiving characteristics (eg, relationship to the patient, caregiving hours, duration, tasks performed, quality of the patient-caregiver relationship, and living situation) are closely related to perceived burden.^45,46,47,48^ Furthermore, we lacked information on the levels of postdischarge support provided to caregivers, which may have influenced our results. Greater levels of caregiver support, tailored to the severity of PICS impairments, could have mitigated burden more effectively compared with lower levels of support provided to caregivers of patients with fewer impairments. The lack of this information limited our ability to interpret the results within the caregiving context. Lastly, with 86% of patients being White and 94% non-Hispanic, the underrepresentation of racial and ethnic minority groups limits the generalizability of our findings.

## Conclusions

In our secondary analysis of a clinical trial, patient PICS and caregiver burden were associated at concurrent time points but not associated with each other over time. Our findings suggest promising directions for future research into the interplay between PICS and caregiver burden in recovery after ICU discharge considering their recognized interdependence.

## Notes

Supplement 1.
Trial Protocol and Statistical Analysis Plan for Parent Study

Supplement 2. **eTable 1. **Bivariate associations among 3-month outcomes and individual characteristics **eTable 2. **Bivariate associations among 12-month outcomes and individual characteristics

Supplement 3.
Data Sharing Statement
