# Supplementary material for: Post–Intensive Care Syndrome and Caregiver Burden: A Post Hoc Analysis of a Randomized Clinical Trial
Source: JAMA Netw Open. 2025 Apr 8;8(4):e253443. doi: 10.1001/jamanetworkopen.2025.3443 (PMC11979734; doi:10.1001/jamanetworkopen.2025.3443)
Supplement: Supplement 2. — eTable 1. Bivariate associations among 3-month outcomes and individual characteristics eTable 2. Bivariate associations among 12-month outcomes and individual characteristics [file jamanetwopen-e253443-s002.pdf]

Supplemental Online Content

Ahn S, LaNoue M, Su H, et al. Post–intensive care syndrome and caregiver burden: a post -hoc analysis of a randomized clinical trial. *JAMA Netw Open*. 2025;8(4):e253443. doi:10.1001/jamanetworkopen.2025.3443

eTable 1. Bivariate associations among 3-month outcomes and individual characteristics

eTable 2. Bivariate associations among 12-month outcomes and individual characteristics

This supplemental material has been provided by the authors to give readers additional information about their work.

© 2025 Ahn S et al. JAMA Network Open.

eTable 1. Bivariate associations among 3-month outcomes and individual characteristics

	Age	Sex	Charlson Comorbidity	IQCODE	Frailty	ADL	FAQ	TICS	PCL-C	CB
**Age**	1.000	NR	NR	NR	NR	NR	NR	NR	NR	NR
**Sex**	.054 (.518)	1.000	NR	NR	NR	NR	NR	NR	NR	NR
**Charlson Comorbidity**	.153 (.064)	.688 (.491)	1.000	NR	NR	NR	NR	NR	NR	NR
**IQCODE**	.436 (<.001)	.634 (.526)	.225 (.006)	1.000	NR	NR	NR	NR	NR	NR
**Frailty**	.247 (.002)	1.750 (.080)	.473 (<.001)	.359 (<.001)	1.000	NR	NR	NR	NR	NR
**ADL**	.096 (.246)	.561 (.575)	.070 (.341)	.123 (.140)	.248 (.002)	1.000	NR	NR	NR	NR
**FAQ**	.093 (.260)	.053 (.958)	.079 (.341)	.221 (.007)	.281 (<.001)	.589 (<.001)	1.000	NR	NR	NR
**TICS**	-.138 (.094)	.031 (.975)	-.093 (.259)	-.199 (.016)	-.065 (.433)	-.172 (.036)	-.179 (.029)	1.000	NR	NR
**PCL-C**	-.432 (<.001)	-.921 (.357)	.039 (.637)	-.007 (.934)	.030 (.722)	.207 (.011)	.303 (<.001)	.010 (.900)	1.000	NR
**CB**	-.166 (.044)	-1.661 (.097)	-.029 (.729)	.053 (.524)	-.009 (.914)	.181 (.027)	.328 (<.001)	.053 (.519)	.193 (.018)	1.000

Note. Coefficients (p-value) are Spearman's rho for continuous-to-continuous associations, *t* (p-value) for between-group.
Abbreviation: ADL = Katz Index of Activities of Daily Living; CB = caregiver burden; FAQ = Functional Activities Questionnaire; IQCODE = Informant Questionnaire on Cognitive Decline in the Elderly; PCL = PTSD Checklist-Civilian version; TICS = Telephone Screening of Cognitive Status

© 2025 Ahn S et al. JAMA Network Open.

eTable 2. Bivariate associations among 12-month outcomes and individual characteristics

	Age	Sex	Charlson comorbidity	IQCODE	Frailty	ADL	FAQ	TICS	PCL-C	CB
**Age**	1.000	NR	NR	NR	NR	NR	NR	NR	NR	NR
**Sex**	.054 (.518)	1.000	NR	NR	NR	NR	NR	NR	NR	NR
**Charlson comorbidity**	.153 (.064)	.688 (.491)	1.000	NR	NR	NR	NR	NR	NR	NR
**IQCODE**	.436 (<.001)	.634 (.526)	.225 (.006)	1.000	NR	NR	NR	NR	NR	NR
**Frailty**	.247 (.002)	1.750 (.080)	.473 (<.001)	.359 (<.001)	1.000	NR	NR	NR	NR	NR
**ADL**	.105 (.203)	1.060 (.289)	.118 (.152)	.247 (.003)	.209 (.011)	1.000	NR	NR	NR	NR
**FAQ**	.053 (.525)	.588 (.557)	.045 (.587)	.286 (<.001)	.203 (.014)	.645 (<.001)	1.000	NR	NR	NR
**TICS**	-.117 (.155)	-1.003 (.316)	-.093 (.260)	-.170 (.040)	-.133 (.107)	-.192 (.020)	-.346 (<.001)	1.000	NR	NR
**PCL-C**	-.351 (<.001)	-.179 (.858)	-.092 (.264)	-.124 (.137)	-.020 (.809)	.359 (<.001)	.366 (<.001)	-.140 (.091)	1.000	NR
**CB**	-.162 (.049)	-1.367 (.172)	.166 (.043)	.109 (.189)	.100 (.228)	.306 (<.001)	.268 (<.001)	.045 (.590)	.259 (.001)	1.000

Note. Coefficients (p-value) are Spearman's rho for continuous-to-continuous associations, *t* (p-value) for between-group.
Abbreviation: ADL = Katz Index of Activities of Daily Living; CB = caregiver burden; FAQ = Functional Activities Questionnaire; IQCODE = Informant Questionnaire on Cognitive Decline in the Elderly; PCL = PTSD Checklist-Civilian version; TICS = Telephone Screening of Cognitive Status

© 2025 Ahn S et al. JAMA Network Open.
