# Supplementary material for: Post–Intensive Care Syndrome and Caregiver Burden: A Post Hoc Analysis of a Randomized Clinical Trial
Source: JAMA Netw Open. 2025 Apr 8;8(4):e253443. doi: 10.1001/jamanetworkopen.2025.3443 (PMC11979734; doi:10.1001/jamanetworkopen.2025.3443)
Supplement: Supplement 3. — Data Sharing Statement [file jamanetwopen-e253443-s003.pdf]

Data Sharing Statement

Ahn. Post–Intensive Care Syndrome and Caregiver Burden. *JAMA Netw Open*. Published April 08, 2025. doi:10.1001/jamanetworkopen.2025.3443

Data

Additional Information: ClinicalTrials.gov ID: NCT01211522; https://clinicaltrials.gov/study/NCT01211522
Data available: Yes
Data types: Deidentified participant data, Data dictionary
How to access data: Deidentified data and the data dictionary will be shared with approval from the MIND-USA Steering Committee and a signed data access agreement. All requests should be sent to timothy.girard@pitt.edu.
When available: With publication

Supporting Documents

Document types: None

Additional Information

Who can access the data: Researchers whose proposed use of the data has been approved
Types of analyses: For a specified purpose
Mechanisms of data availability: With a signed data access agreement
